# Dr. Otto Schmidt's Specific Treatment of Cancer

**Published:** 1903-11-14

**Authors:** 


### Hospital Clinics and Medical Progress.

### DR. OTTO SCHMIDT'S SPECIFIC TREATMENT OF CANCER.

At a meeting of the Abernethian Society held at St. Bartholomew's Hospital last week, Dr. Jossé Johnson read a paper on this subject.

Dr. Johnson said that for some time he had been at Cologne with Dr. Schmidt watching his methods and studying the new immunity treatment for cancer. Dr. Schmidt had discovered a method of cultivating a parasite which did not differ from that described in the researches of Gaylord, Plimmer, Russell, and Schuler, and which he obtained from fresh cancerous tumours. This parasite grew in the anaplastic cell, and irritating it caused division and multiplication and finally destruction of the surrounding tissue. Parts of the new growth thus formed and containing the parasite were shed into the veins and lymph channels and carried to other parts of the body, there reproducing a similar irritative tissue division and change. The prime cause was the parasite, and all successful treatment must be directed towards the destruction of this irritative micro-organism; therefore the remedy must be in the nature of an anti-parasitic not an antitoxin.

Dr. Schmidt had succeeded in the year 1902, after 10 years of experiment, in cultivating a pure culture of this organism, and had caused with it a new malignant growth in two white mice, and he recultivated the same parasite from the artificially-produced cancer.

Three factors seemed necessary to cause a cancer: (1) the parasite, (2) the anaplastic cell, and (3) a suitable environment. So it was found difficult to cause artificially a new growth in any one of the animals selected for experiment. From one single parasitic cell during cultivation microscopical preparations of all the various described phases of the cancer parasite had been obtained. The treatment consists in rendering the patient resistant to the organism either (1) by successive small injections of a sterilized culture 48 hours old; the injection being made into loose cellular tissue of the body remote from the growth, or (2) by injecting the serum from an animal rendered immune by similar injections to those described in the first method. An extract of greater potency can be made by subjecting the culture to extreme cold, using liquid air, and thus breaking up the parasitic envelopes, and afterwards filtering under pressure according to the method of Büchner. The first dose should be $\frac{1}{100}$ milligramme of the culture dissolved in 20 centigrammes of ·5 per cent. carbolic acid solution, on successive days $\frac{1}{10}$, $\frac{1}{5}$, and 1 milligramme are injected. After the first injection there is an intense reaction in and around the tumour and in all the parts which are the seat of metastatic deposits, the temperature rises, though rarely above 102°, and then falls to normal as the treatment continues. Early cancer reacts most markedly and there may be no reaction at first if the case has gone on to the stage of cancerous cachexia.

There is much tenderness, especially in the region of the metastatic deposits, and the pain is often intense. Local rise of temperature is noticed and the skin if involved may become œdematous. Later these signs recede and as the treatment proceeds the tumour shrivels and finally becomes a small fibrous nodule. Twenty-nine cases had been treated comprising carcinoma of the uterus, breast, tongue, abdomen, cheek, etc., and one sarcoma of the thigh. Twenty-eight of these reacted and in all the growth progressively diminished in size as the treatment proceeded. In the twenty-ninth case there was no reaction even when six times the full dose of 1 milligramme had been used. This proved to be a case of ovarian cystoma which was successfully treated by ovariotomy. One hundred and forty single observations were recorded. All cases of cancer reacted; no case which proved to be non-malignant showed any reaction whatsoever. Dr. Johnson then described two cases which he had seen treated.

In the first of these a primary carcinoma of the breast had been removed with the axillary glands, but recurrence was found in the scalp and the supra- and infra-clavicular fossæ. These growths almost disappeared in three months of treatment, and the fibroid contraction of the gland above the clavicle causing much pain it was removed, and microscopic examination showed only a mass of fibrous tissue with a few cancer cells in one part, which were undergoing fatty degeneration.

The second case was one of sarcoma of the thigh. The tumour in this case also became larger, softer and very tender at first, subsequently decreasing in size rapidly, and had almost disappeared at the sixth week of treatment.

One case of encephaloid carcinoma of the breast was successfully treated. In a case of inoperable cancer of the tongue and floor of the mouth the ulcer ceased to be foul after three or four days' treatment, and pieces of slough came away from the edge of the growth, the whole decreasing in size and eventually becoming cicatrized over. The contraction of scar tissue in a patient with extensive pelvic carcinoma resulted in intestinal obstruction which was relieved by colotomy. The scarring is the chief drawback to this treatment. The lecturer spoke only of *treatment*, time alone would show whether a cure had been effected in these cases. Either of the methods described was equally good, the first or active immunisation was more rapid than the passive treatment by the injection of immunised serum. No ill effects had been noticed except the painful reaction and the result of contracting fibrous tissue.

